# Chromosome-level genome of Tibetan naked carp (*Gymnocypris przewalskii*) provides insights into Tibetan highland adaptation

**DOI:** 10.1093/dnares/dsac025

**Published:** 2022-07-21

**Authors:** Fei Tian, Sijia Liu, Bingzheng Zhou, Yongtao Tang, Yu Zhang, Cunfang Zhang, Kai Zhao

**Affiliations:** Qinghai Provincial Key Laboratory of Animal Ecological Genomics, Key Laboratory of Adaptation and Evolution of Plateau Biota, Northwest Institute of Plateau Biology, Chinese Academy of Sciences, Xining, Qinghai, China; University of Chinese Academy of Sciences, Beijing, China; Qinghai Provincial Key Laboratory of Animal Ecological Genomics, Key Laboratory of Adaptation and Evolution of Plateau Biota, Northwest Institute of Plateau Biology, Chinese Academy of Sciences, Xining, Qinghai, China; Qinghai Provincial Key Laboratory of Animal Ecological Genomics, Key Laboratory of Adaptation and Evolution of Plateau Biota, Northwest Institute of Plateau Biology, Chinese Academy of Sciences, Xining, Qinghai, China; University of Chinese Academy of Sciences, Beijing, China; Qinghai Provincial Key Laboratory of Animal Ecological Genomics, Key Laboratory of Adaptation and Evolution of Plateau Biota, Northwest Institute of Plateau Biology, Chinese Academy of Sciences, Xining, Qinghai, China; Henan Normal University, Xinxiang, China; Qinghai Provincial Key Laboratory of Animal Ecological Genomics, Key Laboratory of Adaptation and Evolution of Plateau Biota, Northwest Institute of Plateau Biology, Chinese Academy of Sciences, Xining, Qinghai, China; University of Chinese Academy of Sciences, Beijing, China; Qinghai Provincial Key Laboratory of Animal Ecological Genomics, Key Laboratory of Adaptation and Evolution of Plateau Biota, Northwest Institute of Plateau Biology, Chinese Academy of Sciences, Xining, Qinghai, China; State Key Laboratory of Plateau Ecology and Agriculture, Qinghai University, Xining, Qinghai, China; Qinghai Provincial Key Laboratory of Animal Ecological Genomics, Key Laboratory of Adaptation and Evolution of Plateau Biota, Northwest Institute of Plateau Biology, Chinese Academy of Sciences, Xining, Qinghai, China

**Keywords:** *Gymnocypris przewalskii*, genome, whole-genome duplication, gene family expansion, adaptation

## Abstract

*Gymnocypris przewalskii*, a cyprinid fish endemic to the Qinghai-Tibetan Plateau, has evolved unique morphological, physiological and genetic characteristics to adapt to the highland environment. Herein, we assembled a high-quality *G. przewalskii* tetraploid genome with a size of 2.03 Gb and scaffold N50 of 44.93 Mb, which was anchored onto 46 chromosomes. The comparative analysis suggested that gene families related to highland adaptation were significantly expanded in *G. przewalskii*. According to the *G. przewalskii* genome, we evaluated the phylogenetic relationship of 13 schizothoracine fishes, and inferred that the demographic history of *G. przewalskii* was strongly associated with geographic and eco-environmental alterations. We noticed that *G. przewalskii* experienced whole-genome duplication, and genes preserved post duplication were functionally associated with adaptation to high salinity and alkalinity. In conclusion, a chromosome-scale *G. przewalskii* genome provides an important genomic resource for teleost fish, and will particularly promote our understanding of the molecular evolution and speciation of fish in the highland environment.

## 1. Introduction

The Qinghai-Tibetan Plateau (QTP) is characterized by an extreme environment with low temperature, hypoxia, strong UV radiation and limited food resources. Native species of the QTP have undergone significant genetic, physiological and morphological changes to adapt to this harsh environment.^1–4^ Schizothoracinae is the exclusive Cyprinidae endemic to the QTP, including 12 genera and over 100 species distributed in the QTP and surrounding area.^5^ It has been reported that schizothoracine fish evolved in response to geographic and eco-environmental transformations in the uplift to the QTP.^6^^,^^7^ Extant species of Schizothoracinae and their altitude distribution reflect the phased uplift of the QTP, thus providing an ideal case for studying the adaptation and diversification of fish under palaeoenvironmental changes. Additionally, several studies revealed the association between the evolutionary history of Schizothoracinae and geomorphological changes in the QTP, which provided biotic evidence for the reconstruction of the paleoelevation of the QTP.^8–11^ Therefore, deciphering the genome of schizothoracine fish will advance our knowledge on the adaptive mechanisms of teleosts to the highland environment, and facilitate the understanding of evolutionary processes of the major drainages in the QTP.

Whole-genome duplication (WGD) is considered one of the driving forces for evolution, and provides genetic materials for evolutionary novelty, species diversification and environmental adaptation.^12–14^ Most cyprinid fish experienced an additional round of WGD after the teleost-specific WGD,^15^ and varied in their ploid level, ranging from diploids (2n = 50) to high polyploids (2n = 470).^16–18^ As a sub-family of Cyprindae, polyploidization was observed in all examined schizothoracine species based on karyotype examinations.^18–20^ The phylogenetic analyses showed that a single lineage of the nuclear marker in Schizothoracinae compared with two paralogs in allotetraploid Cyprinini, which indicated possible autotetraploidizaition in schizothoracine fish.^19^^,^^21^*Gymnocypris przewalskii* is a representative species in Schizothoracinae, which resides in Lake Qinghai, the largest inland lake with high salinity and alkalinity (pH up to 9.2, ∼14‰ salinity and altitude of ∼3,200 m) in China.^22^ As a primary food source for migratory birds from Siberia, *G. przewalskii* plays a crucial role in maintaining the stability of ecological processes and biodiversity of the QTP.^23^^,^^24^

The polyploid genomes of Schizothoracinae complicate the sequencing and assembly of a high-quality genome. Although the genome of *G. przewalskii* has been reported (preprint), the assembly information has not been released and the adaptative mechanism has not been comprehensively discussed.^25^ By combining PacBio long-read sequencing, Illumina short-read sequencing and Hi-C technology, we present a chromosome-scale *G. przewalskii* genome, which can serve as the reference genome for schizothoracine fish. The comparative genomics analyses were conducted between *G. przewalskii* and related cyprinid fish to characterize the gene family evolution and WGD in *G. przewalskii*. The high-quality genome of *G. przewalskii* provided new insights into the evolution, speciation and diversification of teleosts, which would advance our understanding on the adaptive mechanisms at the genomic level.

## 2. Materials and methods

### 2.1. Field investigation and ethics statement

A female *G. przewalskii* was net-captured in Lake Qinghai. The fieldwork was authorized and supervised by the Qinghai Provincial Bureau of Fishery. All animal experiments were conducted following the procedures described in the ‘Guidelines for animal care and use’ manual approved by the Animal Care and Use Committee, Northwest Institute of Plateau Biology, Chinese Academy of Sciences.

### 2.2. Genome survey

Two methods were applied to predict the genome size of *G. przewalskii*, including flow cytometry and the *k*-mer method. For flow cytometry, blood samples (0.2–0.5 ml) were centrifuged at <500 (rpm) to obtain red blood cells. Red blood cells were fixed in 70% ethanol at 4°C, and stained with CyStain UV Precise P (Partec, Germany) for 30 min at 4°C. The cell number was counted and reached over 1 × $10^{4}$. Samples and controls were run in CyFlow Space (Partec). A chicken blood sample (2C = 2.30 pg/N) was used as a control.^26^ Second, the blood sample was collected from a female *G. przewalskii*, which was used for the *k*-mer method and subsequent *de novo* sequencing. For the *k*-mer method, genomic DNA was purified from blood samples using a TIANamp Blood DNA Kit (TIANGEN, China) according to the manufacturer’s description. The quantity and quality of DNA was assessed by 1% agarose gel electrophoresis, Qubit^®^ 2.0 Fluorometer (Life Technologies, USA) and Agilent Bioanalyzer 2100 system (Agilent Technologies, USA). Genome survey sequencing was performed to estimate genome size and determine the sequencing depth. Blood DNA was randomly sheared for library construction and sequencing using the Illumina HiSeq X ten platform. The map of *k*-mer distribution with *k *=* *17 (Supplementary Fig. S1) was constructed using Jellyfish (v2.2.6).^27^ The genome size and the heterozygosity were estimated using GenomeScope (v1.0.0).^28^

### 2.3. Library construction and whole-genome sequencing

(Color online) Genomic DNA was sheared to a size range of 15–40 kb, enzymatically repaired and converted into SMRTbell template libraries as recommended by Pacific Biosciences. The resulting SMRTbell templates were sequenced on a PacBio Sequel instrument. A total of 40 SMRT cells were sequenced, producing 225.68 Gb SMRT raw data. Genomic DNA was used to construct paired-end libraries with a 350 bp insert size using a Paired-End DNA Sample Prep kit (Illumina, San Diego, CA). These libraries were sequenced using the Illumina HiSeq X ten platform, producing 192.53 G raw data. Genome sequencing was conducted by Genedenovo Inc. (Guangzhou, China).

### 2.4. *De novo* assembly of the genome using PacBio and Illumina data

Primary contigs were assembled from PacBio long reads by MECAT (v1.0).^29^ Overlaps of long reads were found using the command mecat2pw (parameters: –k 4 –a 2000) and were corrected using the command mecat2cns (parameters: –r 0.9 –c 6 –l 5000). The resulting contigs were polished using more than 100× coverage of Illumina short reads by two rounds of Pilon (version 1.22) with default parameters.^30^

### 2.5. Hi-C assembly

To improve the assembly, we performed an Hi-C experiment. Muscle tissue from female *G. przewalskii* was cross-linked and digested for *de novo* sequencing. DNA fragments with 300–700 bp insert sizes were used for Hi-C library construction^31^ and were sequenced with an Illumina HiSeq 4000 platform. The clean Hi-C reads were first truncated at the putative Hi-C junctions, and the resulting trimmed reads were realigned to the assembly results with BWA-MEM (v0.7.16a-r1181).^32^ Only uniquely aligned paired reads whose mapping quality was more than 20 were used for further analysis. Invalid read pairs, including dangling-end, self-cycle, relegation and dumped products, were filtered by HiC-Pro (v2.8.1).^33^ The uniquely mapped read pairs were used for scaffolds clustered, ordered and oriented onto chromosomes by ALLHIC (v0.9.8).^34^ The genome-wide Hi-C interaction matrix was generated using ALLHiC_plot program and visualized as heatmap using R. Merqury (v 1.3)^35^^,^^36^ was performed to evaluate the completeness to the genome assembly with parameters of tolerable collision rate of 0.001 and a specific *K*mer of 21.

### 2.6. Genome annotation

We applied RNAmmer^37^ for rRNA prediction, and tRNAscan-SE (v2.0)^38^ for the identification of tRNAs, and miRNAs and siRNAs were identified through the Rfam database.^39^ Repeat sequences were annotated using both homology-based and *de novo* approaches. LTR_FINDER (v1.07)^40^ and MGEscan (v1.1)^41^^,^^42^ were used to identify transposable elements. PILER (v1.0),^43^ RepeatScout (v1.05)^44^ and RepeatModeler (v2.01)^45^ were applied for the *de novo* prediction of repetitive elements, which were merged with the REPBASE database^46^ to construct a repetitive sequence database. The final repetitive sequences of the *G. przewalskii* genome were obtained by prediction using RepeatMasker (v4.0.5)^47^ using the constructed database.

Three strategies were adopted to annotate genes in the *G. przewalskii* genome. First, AUGUSTUS (v3.3.3),^48^ SNAP,^49^ GlimmerHMM (v 3.0.4)^50^ and GeneMark (v4.35)^51^ were applied for *ab initio* gene prediction. Second, proteins from *Danio rerio*, *Ctenopharyngodon idellus*, *Cyprinus carpio*, *Sinocyclocheilus anshuiensis*, *Sinocyclocheilus grahami*, and *Sinocyclocheilus rhinocerous* were aligned to *G. przewalskii* using BLASTX with an e-value of 1e−6, and then gene models were defined using Exonerate (v2.2).^52^ Third, we produced 11 RNA-seq libraries from the gill, kidney, heart and intestine of four *G. przewalskii*. RNA was purified using MiniBEST Universal RNA extraction kit (TaKaRa, China). RNA quality was evaluated by 1% gel electrophoresis, Qubit^®^ 4.0 Fluorometer (Life Technologies), and Agilent Bioanalyzer 2100 system (Agilent Technologies, USA). A total of 1.5 μg RNA was used for library construction based on the manufacturer’s description. Briefly, polyT oligo-attached magnetic beads were used to capture mRNA from total RNA, and then fragmented. The synthesis of first-strand cDNA was carried out using random hexamer primer and MMuLV Reverse Transcriptase (RNase H^−^), and followed by the synthesis of second-strand cDNA. After end repair, sequence adaptors were added. The purified cDNA was amplified using PCR with universal PCR primers and index primers. The library was quantified using Qubit^®^ 4.0 Fluorometer (Life Technologies), which were sequenced on the Illumina HiSeq 4000 platform (Novogene, China). Additionally, 19 RNA-seq datasets of *G. przewalskii* were downloaded from the NCBI Sequence Read Archive (SRA) database (Supplementary Table S16). In total, 30 RNA-seq data were filtered by fastp^53^ to obtain clean data, which were mapped to the *G. przewalskii* genome using HISAT (v2.1.0)^54^ to identify splicing junctions. StringTie (v1.3.4)^55^ was used to obtain transcriptomes using aligned reads. Finally, the outputs from *ab initio* prediction, homology search and RNA-seq were combined as inputs for the MAKER pipeline (v2.3.1)^56^ for protein-coding gene annotation, which were then functionally annotated by searching in the SwissProt, UniProt, NCBI non-redundant protein databases, Gene Ontology (GO) and KEGG databases using BLASTP (E value < 1 × 10^−5^) and Blast2GO (v2.3.5).^57^

### 2.7. Identification of orthologous genes and phylogenetic analysis

Protein sequences of nine cyprinid fish, including *D. rerio*, *C. idellus*, *Poropuntius huangchuchieni*, *Oxygymnocypris stewartii*, *Onychostoma macrolepis*, *C. carpio*, *S. anshuiensis*, *S. grahami* and *S. rhinocerous* were downloaded (Supplementary Table S17). First, all-versus-all BLASTP (e-value < 1 × 10^−7^) comparison of all protein sequences was performed between nine cyprinid fish and *G. przewalskii*, and orthologous genes were clustered by OrthoMCL (v1.4).^58^ CAFÉ (v3.1) was applied to identify orthologous genes that had undergone expansion and/or contraction.^59^ GO enrichment analysis was conducted in the expanded gene families in *G. przewalskii* using the clusterProfiler package in R.^60^

Single-copy gene families were identified in 10 cyprinid fish, which were used to construct a phylogenetic tree. MUSCLE^61^ was used to generate a multiple sequence alignment of protein sequences for each single-copy family with default parameters. The alignments of each family were concatenated to a superalignment matrix that was used for phylogenetic tree reconstruction using MrBayes (v3.2).^62^ The divergence time between species was estimated using MCMCtree in PAML (v4.9)^63^ with the options ‘correlated rates clock’ and ‘HKY85’ model. A Markov chain Monte Carlo analysis was run for 1,000,000 generations using a burn-in of 100,000 iterations. The convergence was evaluated using Tracer (v1.7.1) with ESS ≥ 200. The divergence time for *D. rerio* and *C. idella* [51.9–81.8 million years ago (Mya)] as well as *D. rerio* and *C. carpio* (59.3–122.1 Mya) were obtained from the TimeTree database (http://www.timetree.org/) and were used as the calibration points.

### 2.8. Positively selected genes analysis

To find genes that potentially experienced positive selection, the improved branch-site model (model = 2, Nsites = 2) in the codeml program in the PAML package (v4.9) was used to detect signatures of positive selection on individual codons in a specific branch.^64^ For the detection of positively selected genes (PSGs) in *G. przewalskii*, the branch of *G. przewalskii* was used as the foreground branch, and all other branches in the phylogenetic tree were used as background branches.

### 2.9. Whole-genome duplication

The synonymous substitution rate (*K*s) density plot was generated to determine the timing of WGD in *G. przewalskii*. BLASTP was used to obtain the best-match gene pair between *G. przewalskii* and *D. rerio*, *P. huangchuchieni*, *O. macrolepis* and *C. carpio* based on the protein sequences. The protein alignments of gene pairs were then converted into codon comparisons using ParaAT (v2.0),^65^ which were used for *K*s calculation using KaKs Calculator (v2.0).^66^ MCScanX analysis^67^ was carried out to search genome-wide duplications between *G. przewalskii* itself and with *D. rerio* and *C. carpio*. Based on the *K*s rate (*r*) of 3.51 × 10^−9^ per year per nucleotide,^21^^,^^68^^,^^69^ the timing of WGD was estimated according to the formula T = *K*s/2*r*.^68^

### 2.10. Phylogenetic analysis of Schizothoracinae

The reduced representative sequencing data of 13 Schizothoracine species were downloaded from NCBI SRA (Supplementary Table S16). The raw reads were processed using fastp^53^ to obtain clean reads according to the description by Tang *et al.*^70^ Clean reads were mapped to the *G. przewalskii* genome using BWA with the MEM algorithm with parameters of *t* and *k* equivalent to 4 and 32.^32^ Variants were identified using GATK (4.0).^71^ SNPs with a missing rate <0.20 and a minimum allelic frequency >0.05 were considered for phylogenetic tree construction. SNPs were aligned across all the samples, which were applied to ML analysis with *O. stewarti* as the root. An ML tree was constructed using RAxML-HPC2 with the GTRGAMMA model and 1,000 bootstrap replicates.^72^

### 2.11. Inference of effective population size history

We used the pairwise sequentially Markovian coalescent (PSMC) to infer historical changes in effective population size based on sequence data of *G. przewalskii*,^73^ with parameter settings of -p 4 + 25*2 + 4 + 6 -r 4 -t 15 -N 30. For plot settings, we used a generation time of 3 (*G. przewalskii* reaches its sexual maturation at 3) and the mutation rate of 3.51 × 10^−9^ per year per nucleotide.

### 2.12. RNA-seq analysis

RNA-seq data for genome annotation (see Section 2.5) were used to measure the transcription for duplicate genes. To clearly distinguish the expression levels of duplicate copies, clean reads that were uniquely mapped to a single gene without a mismatch were counted to calculate FPKM (Supplementary Table S16). Differentially expressed genes (DEGs) were defined as having an FDR <0.01 and fold change >2.

## 3. Results

### 3.1. Genome assembly and annotation

**Figure 1:**
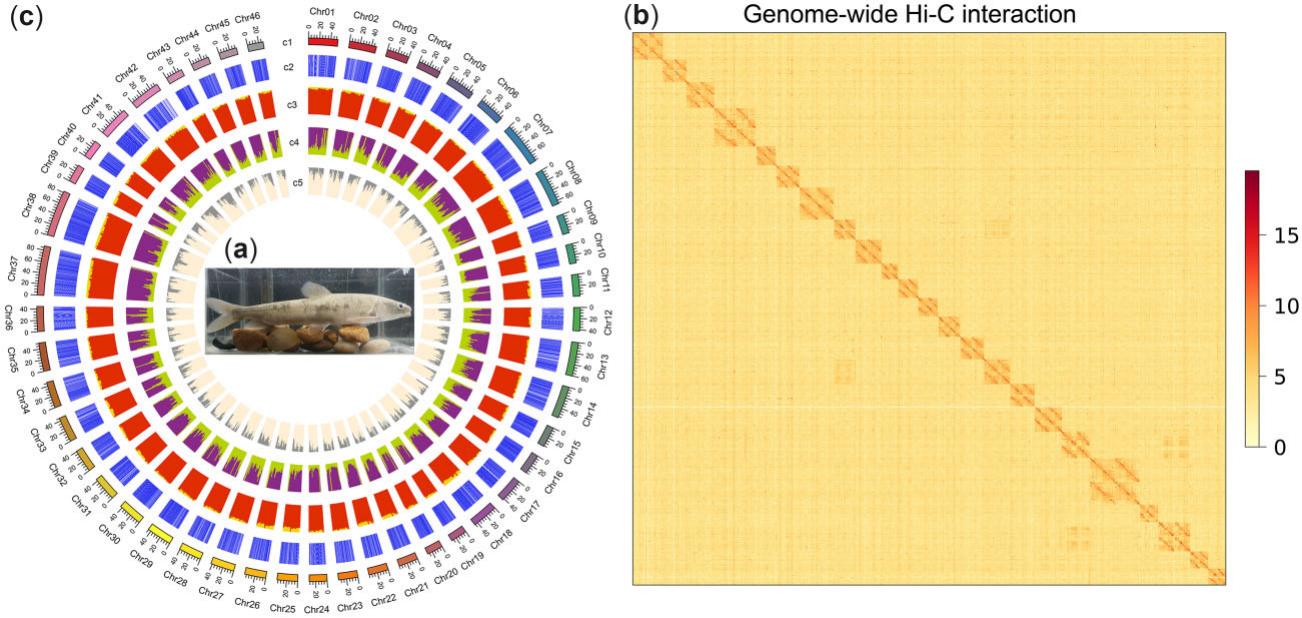
Genome of *G. przewalskii*. (a) Photo of *G. przewalskii* that was net-captured in Lake Qinghai and sequenced in the current study. (b) Chromosomal contact map of *G. przewalskii* using Hi-C data. (c) The *G. przewalskii* genome landscape created using Circos. From outer to inner circles: c1, *G. przewalskii* chromosomes at Mb scale; c2, coding sequences; c3, GC content; c4, repetitive elements drawn in a 100 kb sliding window with a 100 kb step; b5, SNP density in a 100 kb sliding window with a 50 kb step.

The *G. przewalskii* (Fig. 1a) genome size was estimated to be 1.90 Gb by flow cytometry (Supplementary Fig. S1a and b) and 2.19 Gb with heterozygosity of 0.96% by the *k*-mer method (*k *=* *17) (Supplementary Fig. S1c and Table S1). We thus generated sequence data consisting of 230 Gb of PacBio Sequel long reads (∼100x), 110 Gb of Illumina paired-end reads (∼50x) and 350 Gb of Hi-C reads (∼150x). The initial PacBio-based assembly produced 22,550 contigs with an N50 of 136.1 kb (Supplementary Table S2). According to a reported karyotype,^74^^,^^75^ primary contigs were scaffolded into 46 chromosomes, which anchored 96.98% sequences. We developed the final assembly of 2.03 Gb with a scaffold N50 reaching 44.98 Mb (Table 1 and Fig. 1b). This assembly was similar to the *Schizothorax o’connori* genome of 2.07 Gb and larger than the *O. stewartia* genome of 1.85 Gb.^69^^,^^76^ The *G. przewalskii* genome had a GC content of 38.34%. Gaps comprised 0.05% of the genome. The evaluation of the assembly by BUSCO (v3.0.1, vertebrata_odb9) resulted in the identification of 89.4% complete (duplicated: 43.5%; single-copy: 45.9%) and 2.6% fragmented genes from 2,586 vertebrate core single-copy orthologs (Supplementary Table S3). The reference free Merqury evaluation showed a QV (consensus quality) of 31.4 and a completeness of 80.36%, indicating the relatively complete and accurate assembly of the *G. przewalski* genome.

**Table 1:** The statistics of *G. przewalskii* genome assembly

Statistics	Contig	Chromosome	Unanchored
Total number	22,550	46	1,022
Total length (bp)	2,085,351,307	2,029,228,326	63,250,882
Gap (bp)	—	1,084,502	—
Average length (bp)	185,814.94	44,073,952.24	61,889.32
N50 length (bp)	136,081	44,980,694	66,094

Combining prediction, homology searching and RNA-seq data, we obtained 56,397 genes in the *G. przewalskii* genome, among which 50,660 genes (89.83%) were annotated in public databases (Supplementary Table S4). Additionally, non-coding RNAs, including 24,705 tRNAs, 1,653 microRNAs, 2,649 small nuclear RNAs and 2,068 rRNAs, accounted for 0.19% of the genome (Supplementary Table S5). We also identified that repetitive elements occupied 42.89% of the *G. przewalskii* genome by *de novo* searching and searching in repeat databases. DNA transposons (20.49%), long-interspersed nuclear elements (14.05%) and long-terminal repeats (21.31%) were the most abundant repetitive sequence types (Supplementary Table S6). In summary, we generated a chromosome scale of the *G. przewalskii* genome with relatively high completeness and accuracy.

### 3.2. Comparative analysis between *G. przewalskii* and Cyprinidae

**Figure 2:**
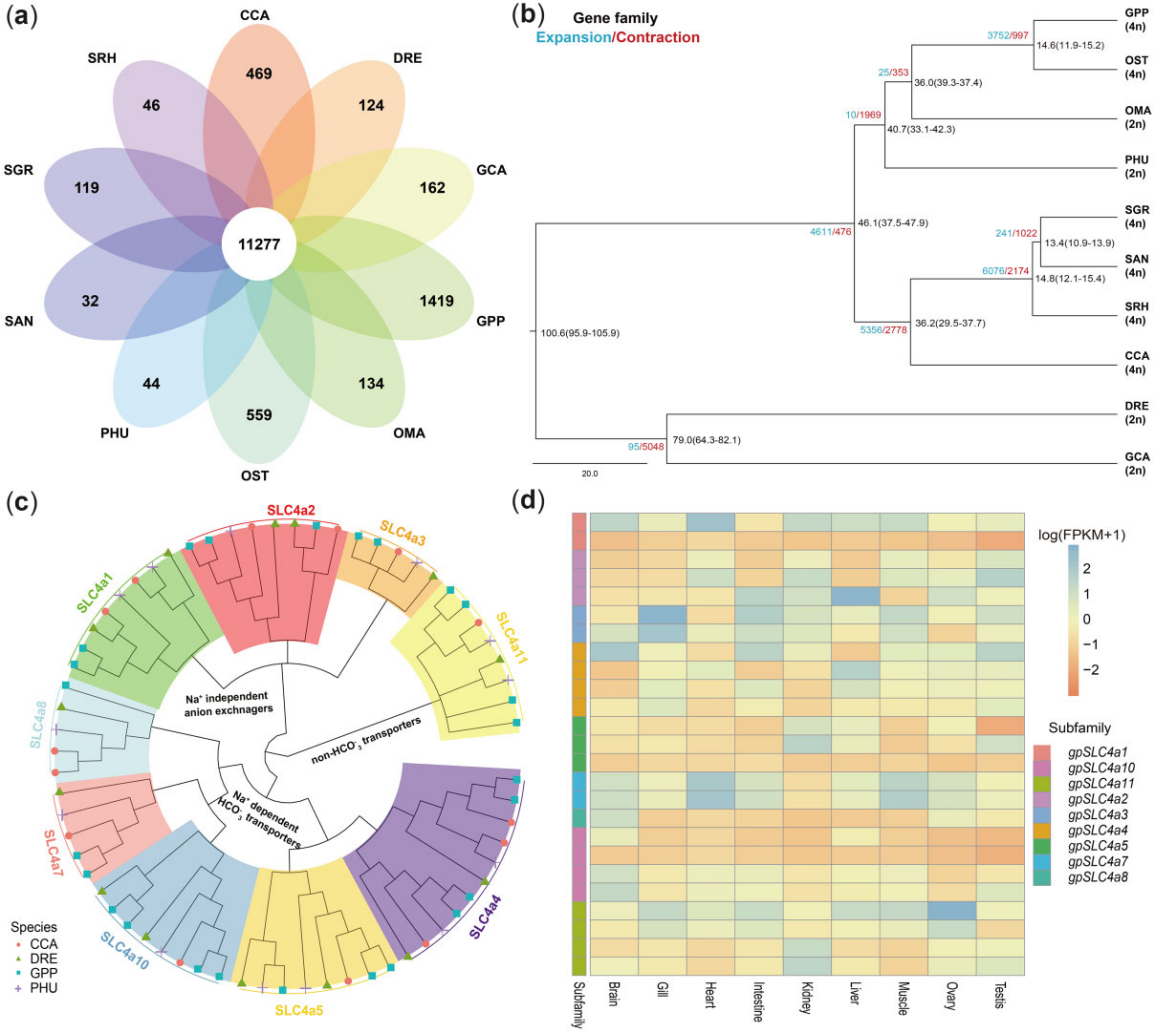
Comparative genomics between *G. przewalskii* and cyprinid fish. (a) Venn diagram of shared and unique gene families among 10 cyprinid fish species. The number represents the number of gene families. GPP, *G. przewalskii*; DRE, *D. rerio*; CCA, *C. carpio*; OST, *O. stewartia*; PHU, *P. huangchuchieni*; OMA, *O. macrolepis*; GCA, *C. idellus*; SGR, *S. grahami*; SAN, *S. anshuiensis*; SRH, *S. rhinocerous*. (b) Phylogenetic tree of 10 cyprinid fish based on single-copy gene families. The species divergence times were estimated and labelled in the branch with 95% HPD. The number of gene family expansions and contractions are labelled in blue and red, respectively. (c) Phylogenetic analysis of the SLC4a gene family in *G. przewalskii* (blue square), *P. huangchuchieni* (purple plus sign), *C. carpio* (red circle) and *D. rerio* (green triangle) based on the protein sequences. (d) Transcription patterns of *gpSLC4a* family members in the brain, gill, heart, intestine, kidney, liver, muscle, ovary and testis (A color version of this figure appears in the online version of this article).

Comparative genomic analysis detected that 11,277 orthologous genes with 204,732 genes were shared by *G. przewalskii* and 9 cyprinid fish (*D. rerio*, *O. stewartii*, *P. huangchuchieni*, *O. macrolepis*, *C. carpio*, *S. grahami*, *S. rhinocerous*, *S. anshuiensis* and *C. idellus*), among which all fish (Fig. 2a;Supplementary Tables S7 and S17). There were 1,419 *G. przewalskii*-specific clusters with 3,468 genes. *Gymnocypris przewalskii* harboured 8,918 expanded and 4,508 contracted gene families, and expanded genes were significantly enriched in GO functions of ion and cation transport and homeostasis (Supplementary Table S8). The phylogenetic tree was constructed using 991 single-copy orthologs identified among *G. przewalskii* and nine cyprinid fish (*D. rerio, C. carpio, S. grahami, S. rhinocerous, S. anshuiensis* and *C. idellus*). The phylogenetic tree indicated that the schizothoracine fish, *G. przewalskii* and *O. stewartia* (4n = 92), had the closest genetic relationship with *O. macrolepis* (2n = 50),^77^ which is currently distributed in the middle reaches of the Yangtze River and the Yellow River in the adjacent areas of the QTP. The split between two schizothoracine fish, *G. przewalskii* in the northeast QTP and *O. stewartii* in the Yarlung Zangbo River of the southern QTP, occurred 14.6 Mya [95% highest posterior density (HPD): 11.9–15.2 Mya] (Fig. 2b), which was related to the early uplift of the QTP.^78^^,^^79^

Interestingly, we noticed that the *SLC4* family was extensively expanded to 25 members in *G. przewalskii*, compared with 14 members in zebrafish, 14 members in common carp and 13 members in *P. huangchuchieni*. The phylogenetic analysis classified *gpSLC4A* members into nine sub-families, all of which were grouped with their counterparts in zebrafish, common carp and *P. huangchuchieni* (Fig. 2c). This classification was consistent with SLC4 transporter types,^80^ including ${\text{Na}}^{+}$-independent anion exchangers (SLC4A1-3), ${\text{Na}}^{+}$-dependent ${\text{HCO}}_{3}^{-}$ transporters (SLC4A4, SLC4A5, SLC4A7, SLC4A8 and SLC4A10) and the non-${\text{HCO}}_{3}^{-}$ transporter (SLC4A11). The expanded members, including *gpSLC4A2-5*, *gpSLC4A7*, *gpSLC4A10* and *gpSLC4A11*, covered each transporter type and displayed more variable expression patterns among tissues (Fig. 2d).

### 3.3. Positively selected genes

Based on single-copy orthologs, we identified 141 PSGs in *G. przewalskii* by comparison with nine cyprinid fish (Supplementary Table S9). Although these PSGs were not significantly enriched in any particular GO terms, we found that the hypoxia-related genes *EPAS1* and *HIF1a* were also detected as PSGs in *G. przewalskii* (Supplementary Table S9), which were shown to contain beneficial mutations and contribute to hypoxia adaptation in many Tibetan species.^1^^,^^3^^,^^4^^,^^81^ Since *G. przewalskii* adapts to the high saline and alkaline environment (12–14‰ salinity) of Lake Qinghai, we paid particular attention to genes for iono- and osmoregulation. Evidence of positive selection was identified in *SLC22a23*, a member of the solute carrier superfamily involved in organic cation transport.^82^ Two positively selected sites were identified in *AQP3*, a gene mainly expressed in the gill and kidney as a water channel function^83^ (Supplementary Fig. S2). Genes in cell junctions were upregulated in *G. przewalskii* under high salinity.^84^ We found a calcium-dependent cell–cell adhesion glycoprotein, *CDH4*, harbours three positively selected sites (Supplementary Fig. S3), which might play an important role in preventing the diffusion of water and/or salts in high salinity. The positively selected sites in genes related to iono- and osmoregulation might lead to structural and functional changes, which would contribute to the adaptation of *G. przewalskii* to high salinity in Lake Qinghai.

### 3.4. Population demography

**Figure 3:**
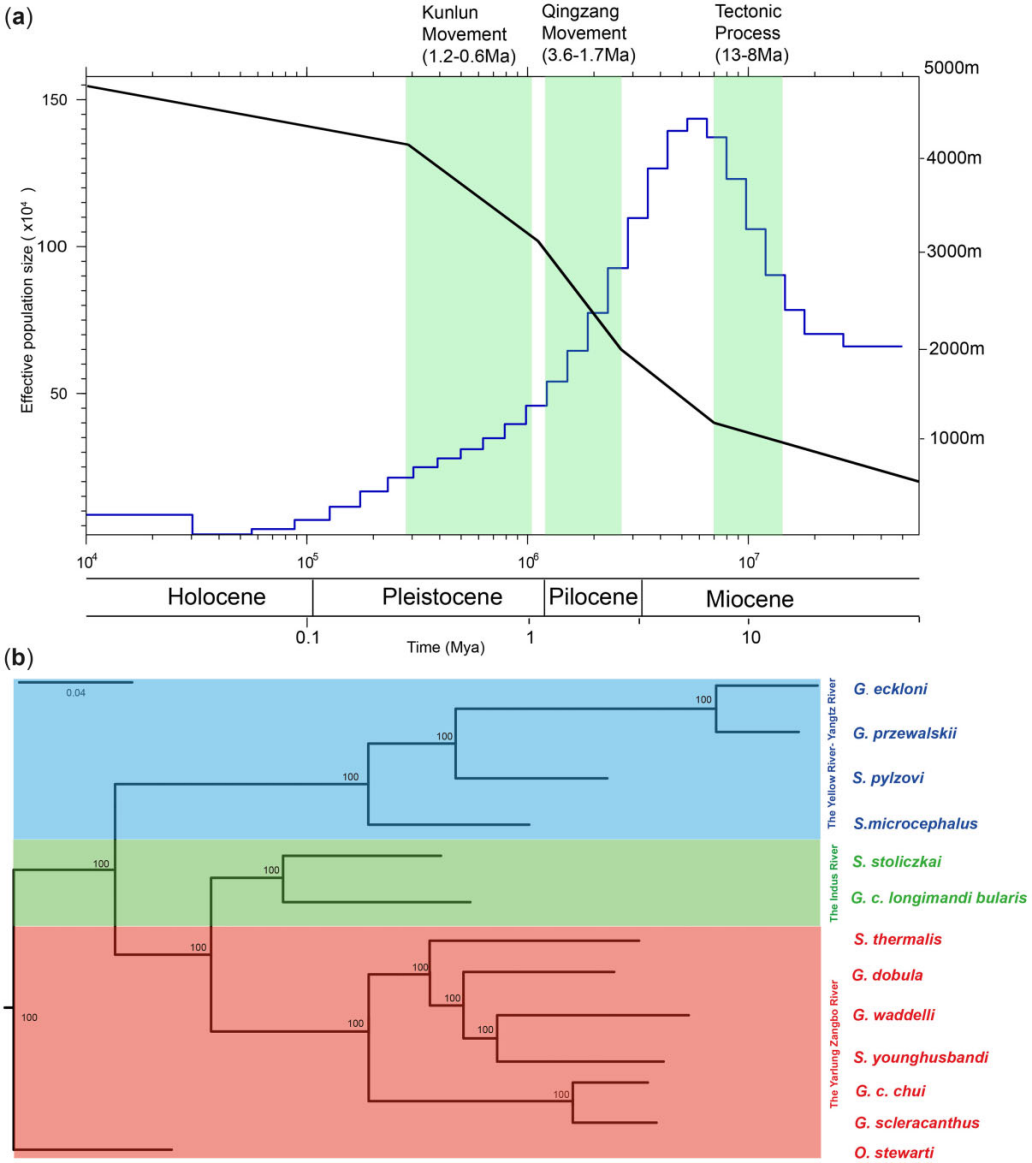
Phylogenomic analysis and dimorphic history. (a) Phylogenetic tree of schizothoracine fish. The phylogenetic tree of 13 schizothoracine fish was inferred by the maximum likelihood (ML) method based on SNPs. Bootstrap support values are labelled in the branch. Fishes from drainages of the Yellow River-Yangtz River, Indus River and the Yarlung Zangbo River are shown in blue, green and red, respectively. (b) Inference of *G. p. przewalskii* population history. The left *y* axis denotes the effective population size of *G. przewalskii* in a time-series manner (blue line). The right *y*-axis represents the approximate altitude in the northeastern QTP according to the estimation from Li *et al.*, Wu *et al.* and Deng *et al.* (black line). The periods of important geographic movements are shaded in green (A color version of this figure appears in the online version of this article).

Population history reconstructed using the PSMC model suggested a strong correlation between the demography of *G. przewalskii* and the upheaval of the QTP (Fig. 3a). First, ancient *G. przewalskii* experienced population expansion (∼13–8 Mya) and reached a maximum effective population size from the middle to late Miocene (∼27–8 Mya). This period was coincident with the early lifting of the QTP (∼25–17 Mya) and the tectonic process occurring 13–8 Mya that steadily lifted the QTP to 1–2 km.^85–87^ Then, the population size appeared to dramatically decline during the Qingzang movement (3.6–1.7 Mya)^78^ and Kunhuang movement (1.2–0.6 Mya),^88^ when the QTP was intensively elevated from ∼2 km to its present altitude.^79^^,^^89^^,^^90^

### 3.5. Phylogenomic reconstruction of Schizothoracinae

The SLAF-seq data of 13 species in Schizothoracinae were obtained from NCBI (Supplementary Table S10), including the *gymnocypris* genus, *schizopygopsis* genus and *oxygymnocypris* genus,^70^ which were aligned to the *G. przewalskii* genome. The mapping ratios ranged from 80.85% in *Schizopygopsis thermalis* (distributed in the southwestern QTP) to 91.57% in *Gymnocypris eckloni* (Supplementary Table S10), which produced 287,222 informative SNPs. The reconstructed phylogenetic tree classified 13 schizothoracine fish into three major clades (Fig. 3b), consistent with their geographic distributions. Species from the genera *Gymnocypris* and *Schizopygopsis* formed polyphyletic relationships, indicating the incongruence between taxonomic classification and molecular phylogeny in Schizothoracinae. This result indicated that the *G. przewalskii* genome could serve as a reference for schizothoracine fish, which could promote our understanding of the taxonomic classification and nomenclature of cyprinid fish.

### 3.6. Genome duplication in *G. przewalskii*

**Figure 4:**
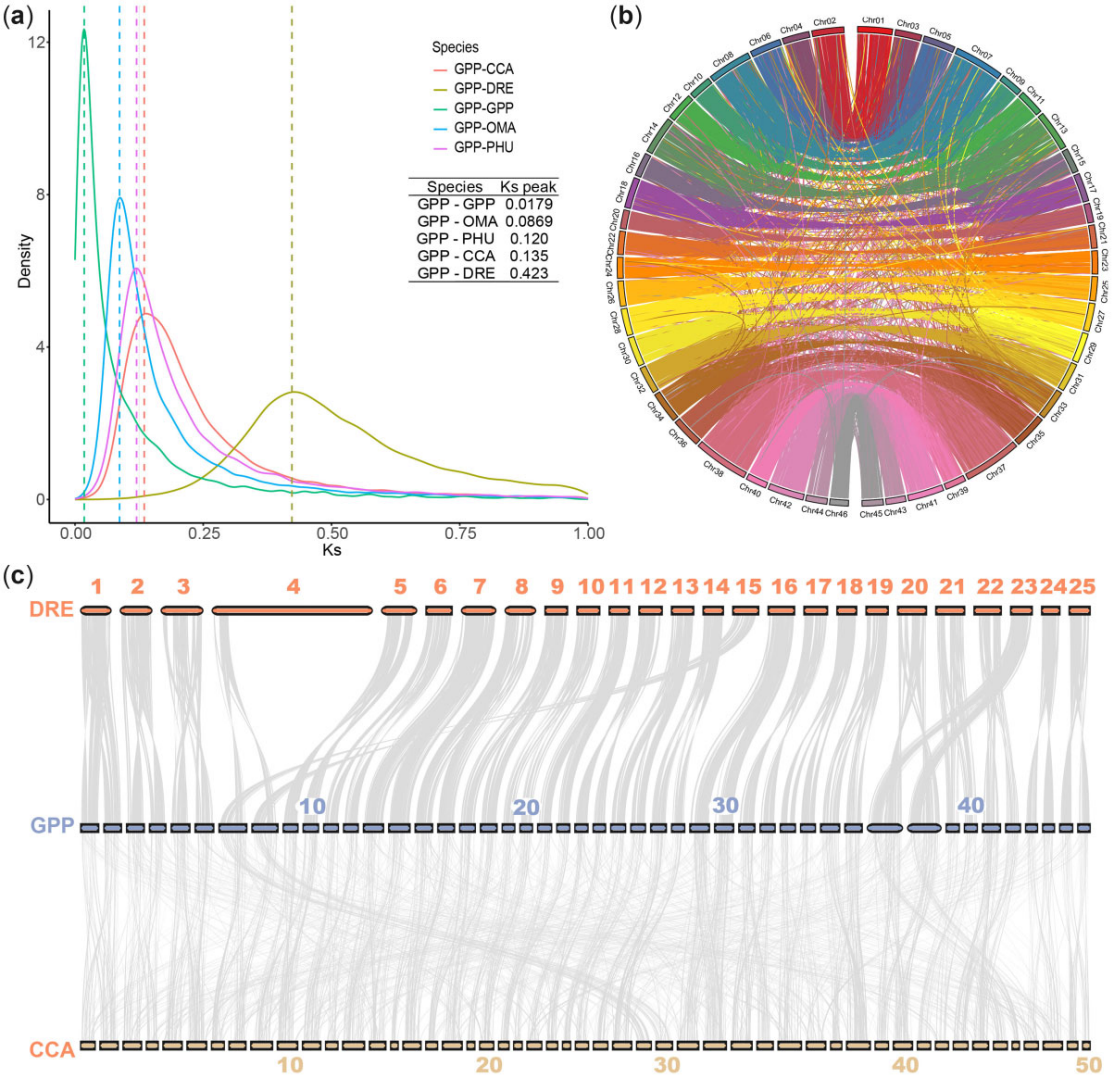
WGD of *G. przewalskii*. (a) Density distribution of *K*s (synonymous substitution rate) of homolog gene pairs of GPP–GPP, GPP–PHU, GPP–OMA, GPP–CCA and GPP–DRE. Dashed lines indicate the peak values in each species pair. GPP, *G. przewalskii*; DRE, *D. rerio*; CCA, *C. carpio*, PHU, *P. huangchuchieni*; OMA, *O. macrolepis*. (b) Chord diagram displaying the synteny of 46 *G. przewalskii* chromosomes on the basis of 754 syntenic blocks, as indicated by lines. The chromosomes are ordered by size, and homologous chromosomes are labelled with the same colour. (c) Collinearity among 46 *G. przewalskii* chromosomes (middle), 50 *C. carpio* chromosomes (bottom) and 25 *D. rerio* chromosomes (top).

WGD is considered one of the driving forces of evolution. Cyprinidae experienced an additional round of genome doubling after the teleost-specific genomic duplication event.^14^^,^^74^^,^^75^ A small *K*s peak of 0.018 was observed in *G. przewalskii*, suggesting that it experienced a recent WGD and is a young tetrapolyploid fish. Based on the *K*s distribution, we determined that WGD in *G. przewalskii* occurred at ∼2.55 Mya based on the *K*s rate of 3.51×10^−9^ per year per nucleotide (Fig.4a), which was close to the WGD of *S. o’connori* at 1.23 Mya.^69^

To identify genes produced by WGD in *G. przewalskii*, we performed intra- and inter-synteny analyses. Homology was clearly observed in *G. przewalskii* chromosome pairs, which revealed 754 collinear blocks covering 55.24% of coding genes (31,156/56,397) (Fig. 4b;Supplementary Table S11). A total of 8.64% of genes (4,870) were classified as singleton genes, which were considered the genes that lost one copy (Supplementary Table S11). Notably, ∼64% of *G. przewalskii* specific genes (3,380/5,280) originated from WGD, suggesting that WGD might also have contributed to *G. przewalskii* speciation. GO enrichment suggested that duplicate genes were overrepresented in pathways related to highland adaptation, such as voltage-gated sodium channel activities (GO:0005248), regulation of respiratory gaseous exchange (GO: 0043576) and cellular response to UV (GO:0034644) (Supplementary Table S12). The comparisons of homologous gene pairs between *G. przewalskii* and zebrafish showed clearly collinear patterns, in which two *G. przewalskii* chromosomes roughly corresponded to one zebrafish chromosome (Fig. 4c;Supplementary Fig. S4a). Since the arm in zebrafish chromosome 4 contained potential regions specific to zebrafish,^91^ we did not observe collinearity with any regions in *G. przewalskii* chromosomes. In total, 11,942 *G. przewalskii* genes had a two-to-one relationship in *D. rerio* (Supplementary Table S13), accounting for 54% of the *G. przewalskii* genome (Supplementary Fig. S4b). These triplet gene pairs were enriched in cold acclimation (GO:0009631), regulation of respiratory gaseous exchange (GO: 0043576) and response to UV (GO:0071493) (Supplementary Table S14), suggesting that WGD in *G. przewalskii* might contribute to its adaptation to the QTP highland. We also identified 4,835 and 4,183 genes in *G. przewalskii* with one-to-one and two-to-one copies in *C. carpio*, respectively (Fig. 4c;Supplementary Fig. S5 and Table S13). We found that 2,944 *G. przewalskii* genes had two-to-one relationship with both *D. rerio* and *C. carpio*, which might be due to allotetraploid in *C. carpio* and autotetraploid in *G. przewalskii*. Genes with two paralogs in *G. przewalskii* but one in *C. carpio* were enriched in GO terms, such as response to caloric restriction (GO:0061771), sequestering of metal ion (GO:0051238) and adaptive thermogenesis (GO:1990845) (Supplementary Table S15). These results indicated that duplicate genes in *G. przewalskii* contributed to highland adaptation.

### 3.7. The evolution and transcription of duplicate gene pairs

The evolutionary outcomes of gene duplication generally fall into three categories: non-functionalization of one copy by degenerative mutations, neofunctionalization of one copy and sub-functionalization of both copies.^92–94^ We analysed the transcription of duplicate pairs in the brain, gill, heart, intestine, kidney, liver, muscle, ovary and testis of *G. przewalskii* (Supplementary Fig. S6 and Table S16), and the DEGs were identified between tissue comparisons. Among 31,156 duplicate genes, 26,036 were transcribed in nine organs. These expressed gene pairs were classified into two categories, either showing equal expression among all tissues (1,424 pairs) or displaying different transcription between at least two tissues (10,322 pairs). In the latter category, 2,515 pairs exhibited tissue-dependent expression patterns of one copy and equal transcription of the other copy. Among the remaining gene pairs (7,807), 7,150 gene pairs showed that two copies had partitioned transcription patterns between at least two tissues (Supplementary Fig. S7). Diverse transcriptional profiles of duplicate pairs highlighted possible neo- or sub-functionalization of duplicate genes in *G. przewalskii*.

## 4. Discussion

As an endemic fish of the QTP, *G. przewalskii* exhibits great adaptation to severe natural conditions, such as high salinity and alkalinity, chronic cold and hypoxia, which represents a remarkable model to study the evolutionary process in the extreme environment. In the present study, we assembled the chromosome-scale genome of *G. przewalskii* (2.03 Gb) with 56,397 protein-coding genes, which were comparable to the tetraploid cyprinid fish, including *C. carpio* (1.83 Gb with 52,610 genes) and schizothoracine fish of *S. o’connori* (2.07 Gb with 43,731 genes) and *O. stewartii* (1.85 with 46,400 genes).^69^^,^^76^^,^^95^ The genome sizes and gene numbers of these tetraploid fish were doubled compared with potential diploid ancestors *P. huangchuchieni* (1.02 Gb with 24,099 genes) and *O. macrolepis* (928 Mb with 24,770 genes), presumably due to WGD (Supplementary Table S18).^77^^,^^96^ The chromosome numbers in schizothoracine fish ranged from 92 to 96, which might result from the fusion of some chromosomes in the potential autotetraploidization.^69^

The origination of Schizothoracinae is still under debate, and their diploid ancestors have not yet been identified.^18^ Based on the genomic data, the phylogenetic analysis suggests that it was evolved from diploid *O. macrolepis* (sub-family Barbinae) that were currently distributed in the middle reaches of the Yangtze River in sub-trophic areas. This result was consistent with fish fossils in the QTP, which were morphologically similar to Cyprinidae in warm regions. This finding was not only significant for the phylogeny and zoogeography of fish, but also had implications for understanding the paleo-elevations of the QTP.^9^ The demographic history of ancient *G. przewalskii* confirmed that the evolution of schizothoracine fish was influenced by environmental changes induced by the uplift of the QTP.^6^ The period of population expansion (∼13 to 8 Mya) was coincident with a pulse of rapid uplift in the northeastern QTP,^97^^,^^98^ which also overlapped with adaptive radiation of the Cyprininae fishes ∼23 to 16 Mya.^18^ A growing body of evidence suggests that QTP uplift leads to extensive eco-environmental alterations, such as the onset of the Asian monsoon and the formation of a temperate continental climate.^9^^,^^79^^,^^89^^,^^99^^,^^100^ The transition to temperate and arid weather might favour the expansion of ancestral *G. przewalskii*, allowing them to replace fishes living in warm and humid areas.^7^ The continuous population declines (from ∼6 to 0.1 Mya) are considered consequences of the comprehensive geological, drainage, climatic and ecosystem changes during the major phase of QTP uplift, which restricted the survival and colonization of *G. przewalskii*.

The impact of WGD on evolution was extensive, providing genomic materials for the origin of evolutionary novelty and facilitating diversification.^13^ The impact of WGD on evolution was extensive, providing genomic materials for the origin of evolutionary novelty and facilitating diversification.^13^ Our analysis suggested that the WGD of *G. przewalskii* occurred at 2.25 Mya, which might confer their adaptive advantages to the environmental changes in the extensive uplift in the Qingzang movement (3.6–1.7 Mya).^89^^,^^101^^,^^102^ Genes preserved post-WGD were not random, and were functionally associated with adaptation to extreme highland environments. Additionally, expression variations within gene pairs may have resulted in enhanced vigour and rapid adaptation to the novel conditions,^103^ allowing ancient *G. przewalskii* to cope better than its diploid relatives with the highland environment. Therefore, the evolutionary mechanisms may have considerably shaped the tetraploid genome of *G. przewalskii* by preserved duplicate genes related to environmental adaptation from being lost. Therefore, deciphering the *G. przewalskii* genome will improve our knowledge on the genome evolution of polyploid fish species during eco-environmental changes.

## 5. Conclusions

In the current study, we generated a high-quality chromosome-level assembly of the *G. przewalskii* genome, providing a valuable genomic resource for comparative analysis across teleost fish. Our analysis underscored the importance of WGD, which could grant *G. przewalskii* adaptive advantages to the environmental changes during the QTP uplift. The extensive genomic changes, including positive selection and gene family expansion, facilitated the evolution of *G. przewalskii* to the extreme aquatic environment in the QTP. Additionally, we demonstrated that our assembly could serve as a reference for schizothoracine fishes, which will decipher their origination and evolution. Conclusively, the availability of the genomic resources of *G. przewalskii* will benefit future evolutionary and speciation studies on cyprinids, particularly regarding molecular adaptation, genomic conservation and phylogeny.

## Supplementary Material

dsac025_Supplementary_Data

## Data Availability

Sequencing raw data of PacBio sequence, Illumina sequence and Hi-C, and whole genome assembly of *G. przewalskii* were deposited in CNGB database under project number CNP0002001. RNA-seq data produced by the present study were deposited under accession numbers SRP284182 and SRP284021. Published *G. przewalskii* RNA-seq data used in this manuscript were deposited in NCBI SRA under accession Nos SRP136464, SRP045343, SRP045396, SRP045343, SRP045396 and SRP254169.
