# Supplementary material for: Chromosome-level genome of Tibetan naked carp (Gymnocypris przewalskii) provides insights into Tibetan highland adaptation
Source: DNA Res. 2022 Jul 21;29(4):dsac025. doi: 10.1093/dnares/dsac025 (PMC9326183; doi:10.1093/dnares/dsac025)
Supplement: dsac025_Supplementary_Data [file dsac025_supplementary_data.zip › Supplementary Figure-0526.docx]

**Supplementary Figure Legends**

**Figure S1. The estimation of *G. przewalskii* genome size. a & b.** Flow cytometry was performed to calculate the genome size of *G. przewalskii* (**a**) and *C. carpio* (**b**) using blood samples. Chicken samples were used as the control. **c.** *K*-mer method was adopted to predict the genome size of *G. przewalskii* before PacBio sequencing.

**Figure S2.** **The positions of positively selected sites in *gpAQP3*.** The black arrows indicate positively selected sites (PSSs). The position of PSSs and *p*-values (in the parentheses) of the likelihood ratio test were listed.

**Figure S3.** **The positions of positively selected sites in *gpCDH4*.** The black arrows indicate the positively selected sites (PSSs). The position of PSSs and *p*-values (in the parentheses) of the likelihood ratio test were listed.

**Figure S4. Synteny between *G. przewalskii* and *D. rerio* genomes. a.** Dot plotting of syntenic genes between *G. przewalskii* and *D. rerio*. *G. przewalskii* chromosomes were listed horizontally, and *D. rerio* chromosomes were listed vertically. **b.** The synteny pattern between *G. przewalskii* and *D. rerio* genes was tested as 2:1. GPP: *G. przewalskii*, DRE: *D. rerio*.

**Figure S5. Synteny between *G. przewalskii* and *C. carpio* genomes. a.** Dot plotting of syntenic genes between *G. przewalskii* and *C. carpio*. *G. przewalskii* chromosomes were listed horizontally, and *C. carpio* chromosomes were listed vertically. **b.** The synteny pattern between *G. przewalskii* and *D. rerio* genes. Because *C. carpio* was allopolyploidy, about half of *G. przewalskii* genes did not show clear syntenic relationship to *C. carpio* genes and *vice versa*. GPP: *G. przewalskii*, CCA: *C. carpio*.

**Figure S6. Transcriptional profiles of duplicate genes in *G. przewalskii***. Two copies of each duplicate pair were plotted in two rows next to each other. Gill, kidney and intestine (short for Int) had 4 biological replicates, and brain, ovary, testis, muscle, liver and heart had 3 replicates.

**Figure S7. The illustration of transcriptional analysis of duplicate genes in *G. przewalskii***.

**Figure S1**

**
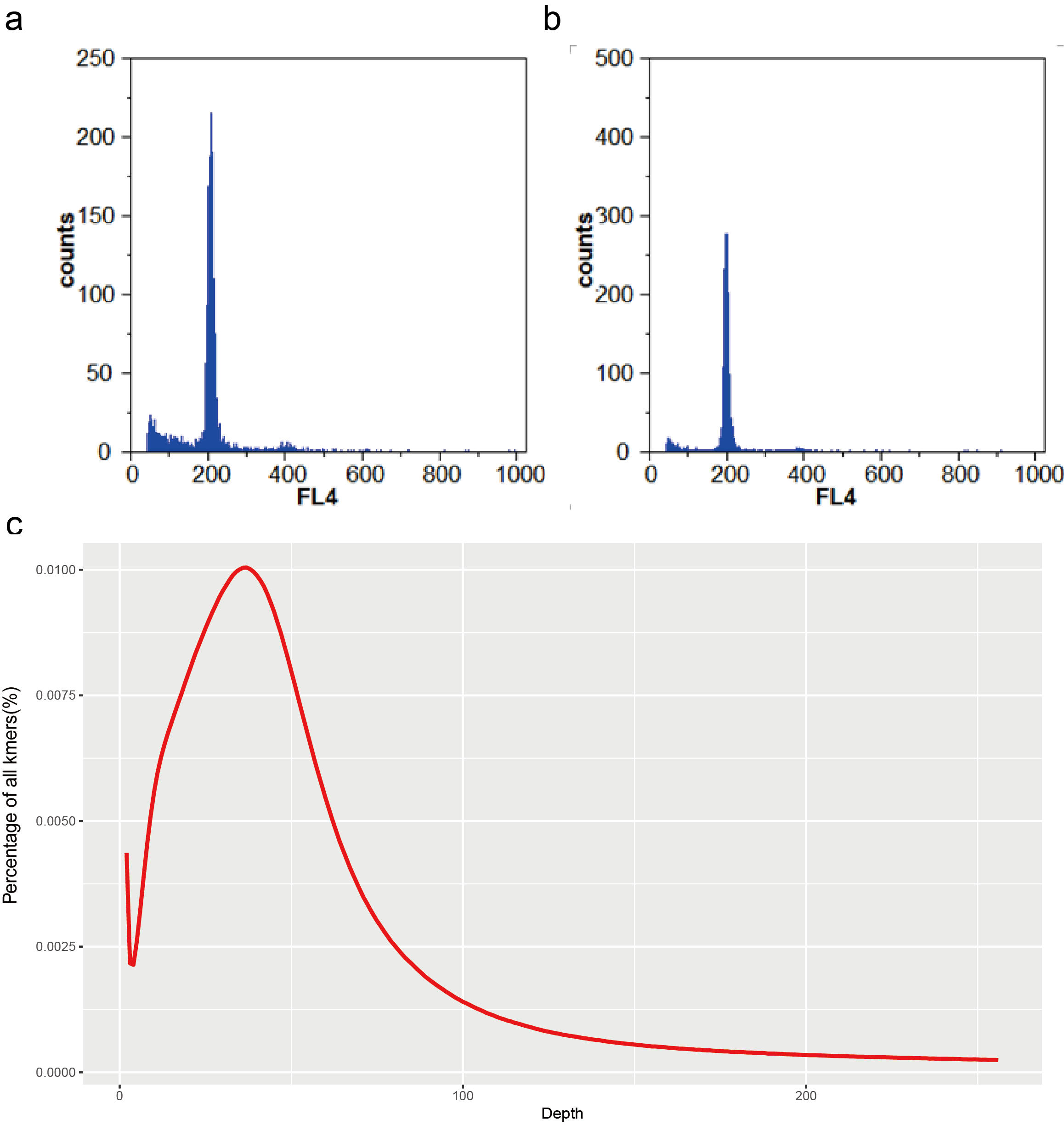
**

**Figure S2**

**
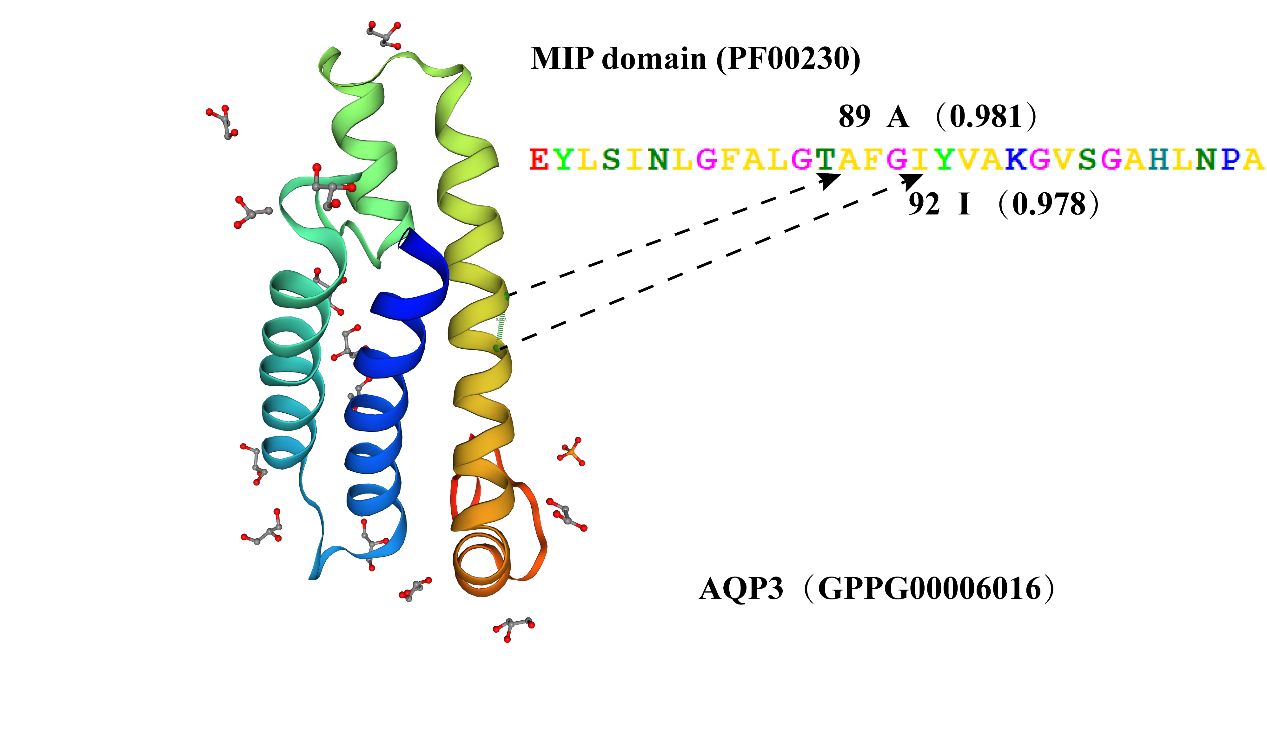
**

**Figure S3**

**
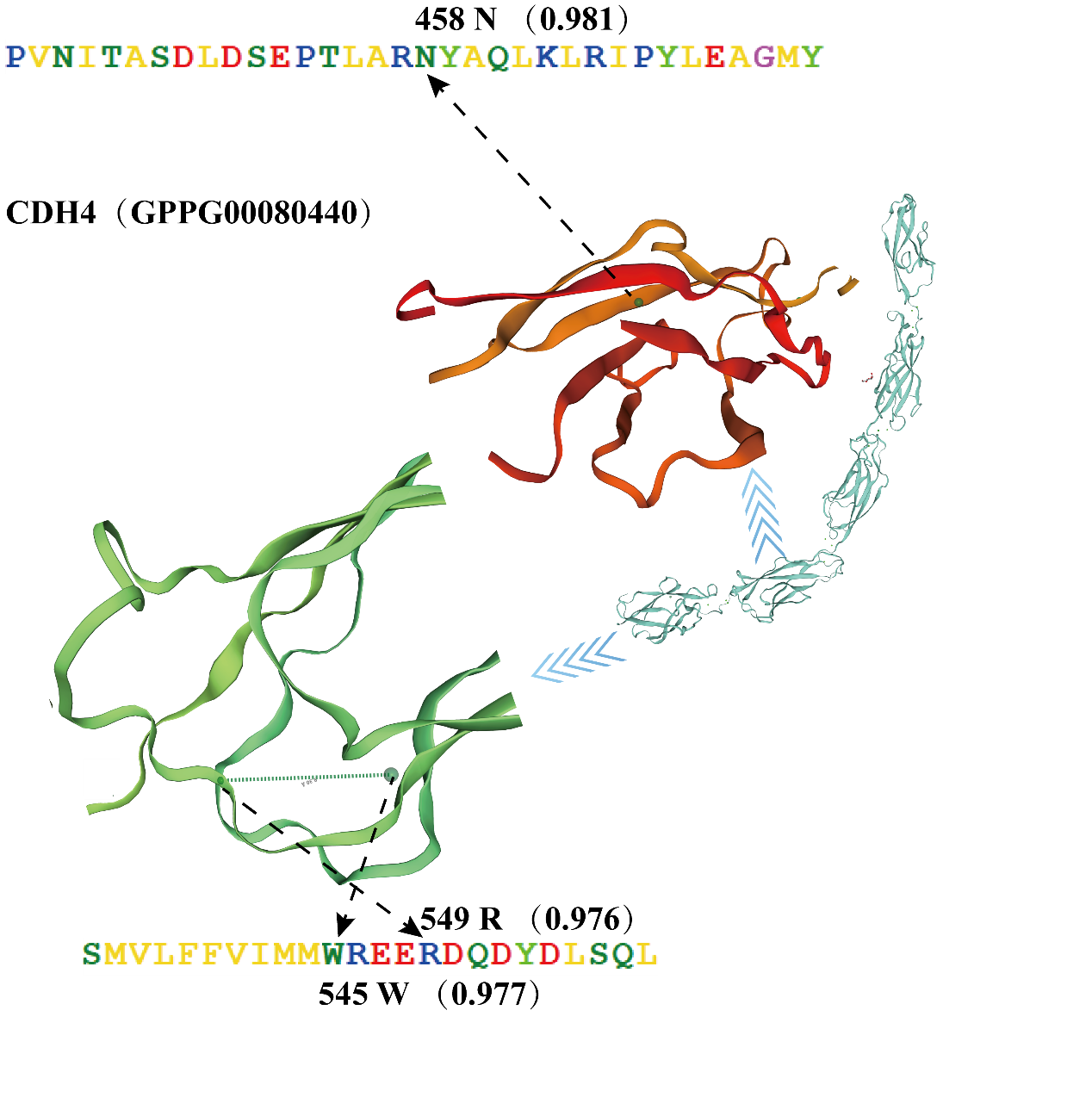
**

**Figure S4**

**
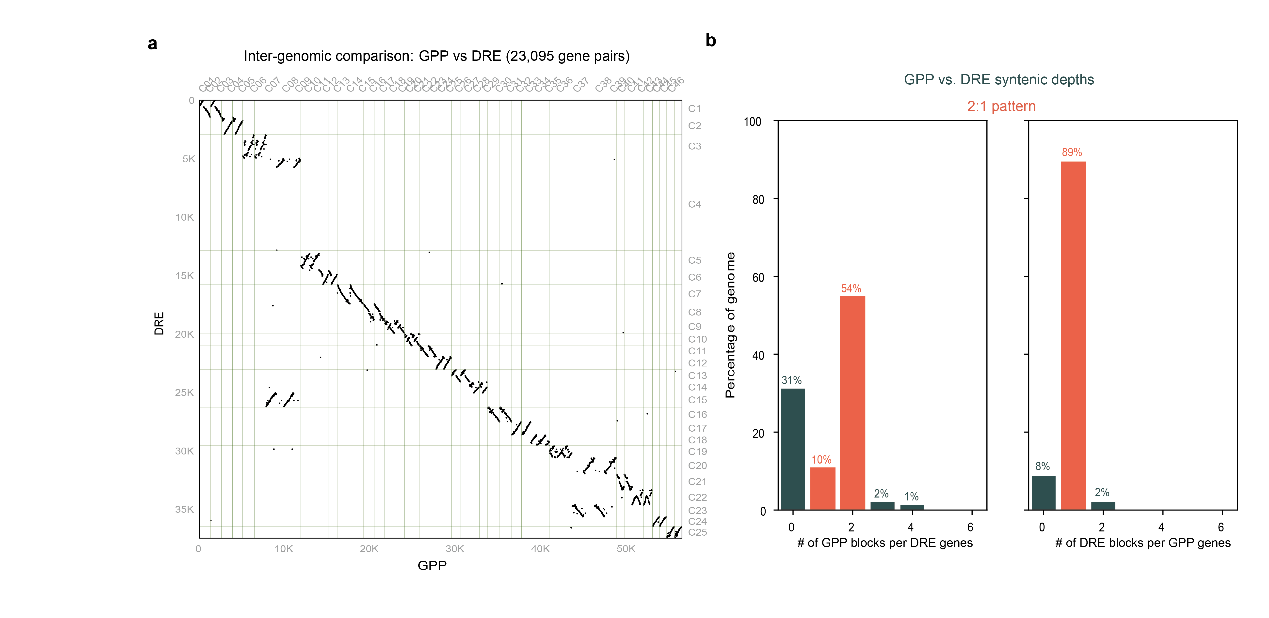
**

**Figure 5**

**
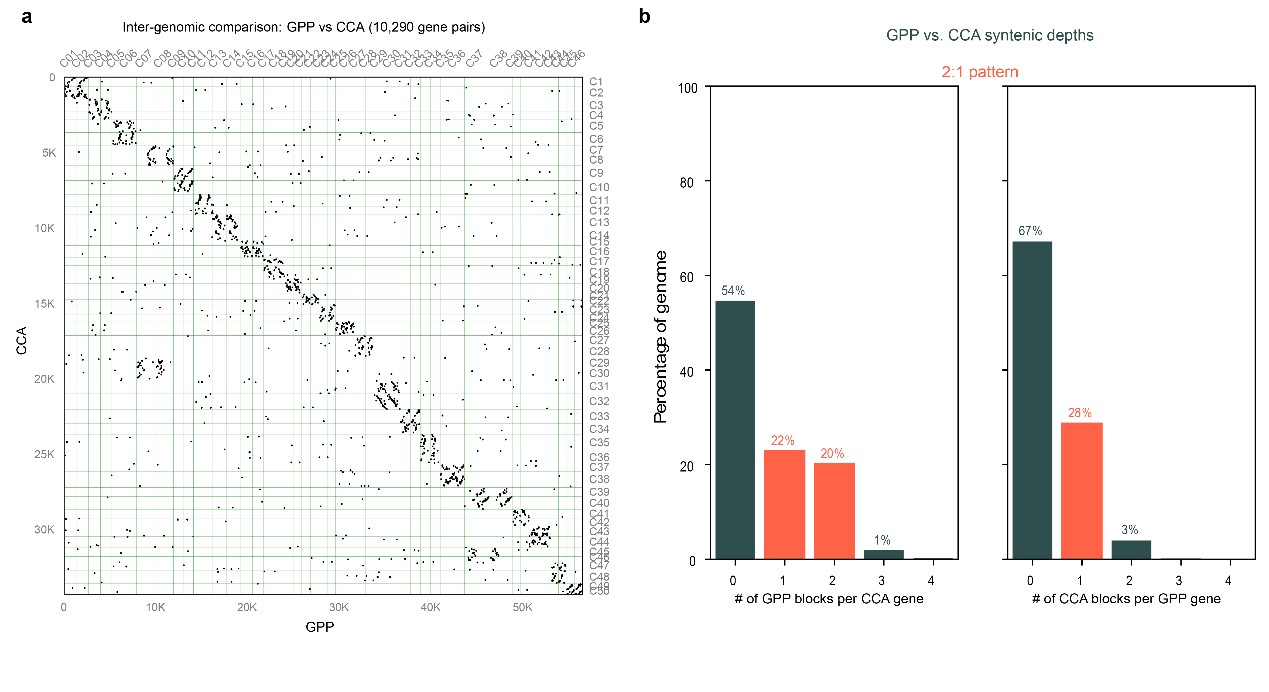
**

**Figure 6**

**
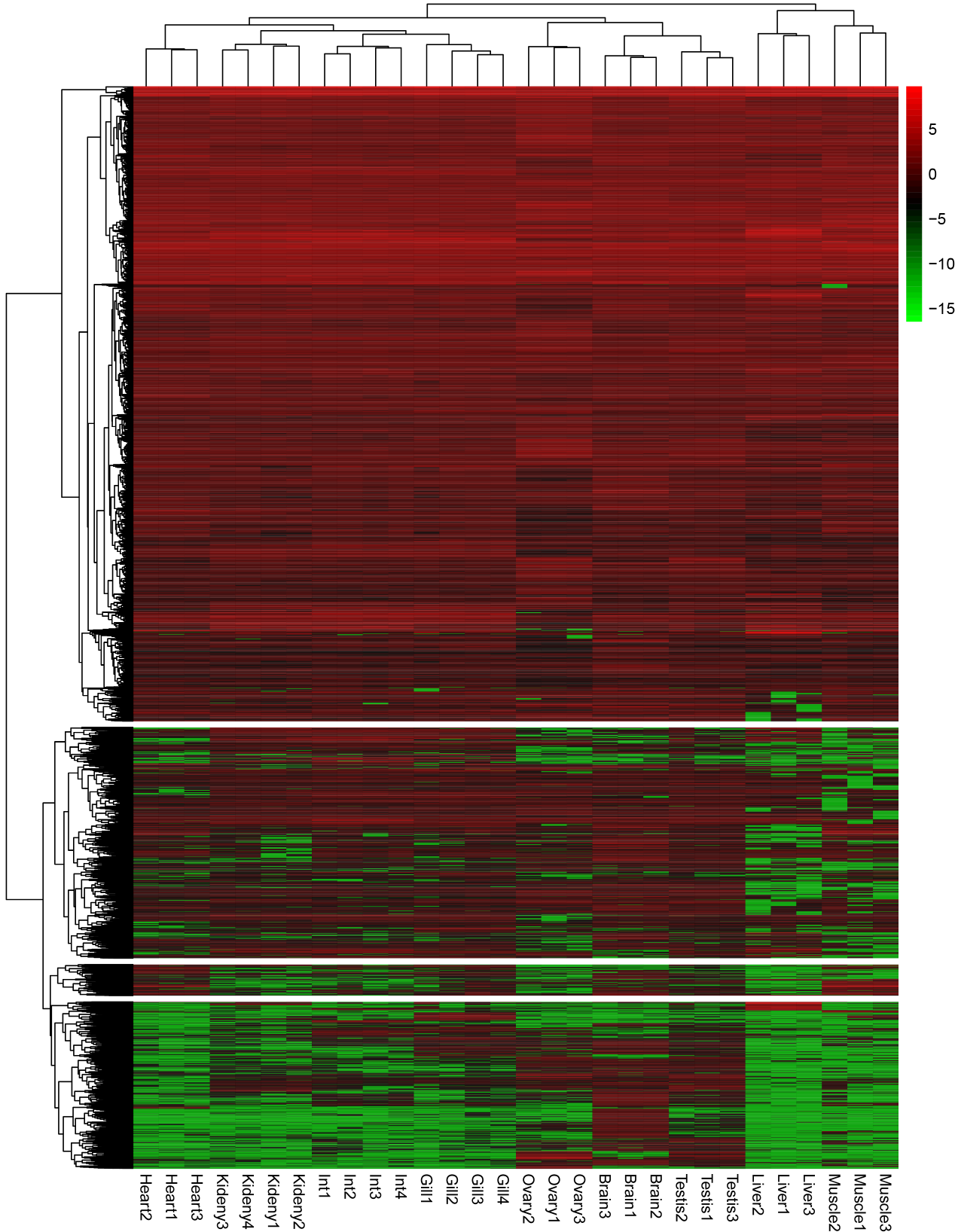
**

**Figure 7**

**
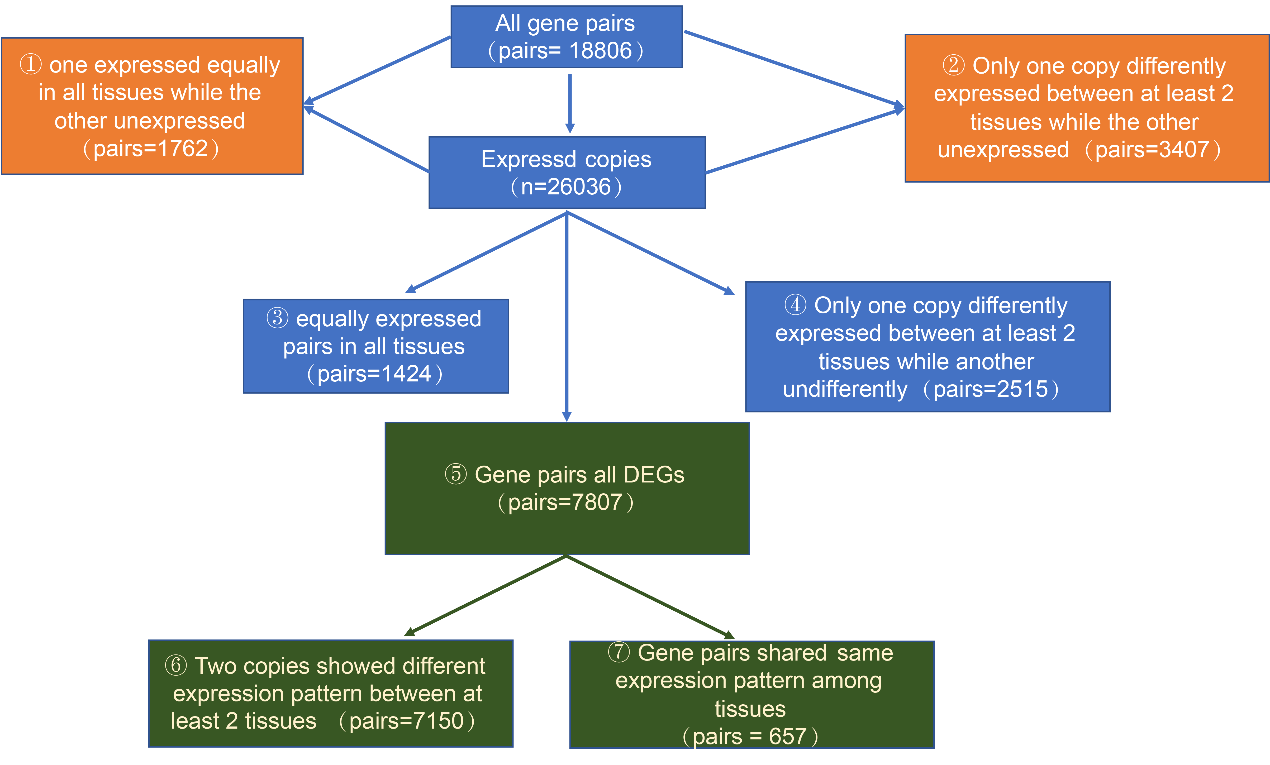
**
